# Gender differences in beliefs about health: a comparative qualitative study with Ghanaian and Indian migrants living in the United Kingdom

**DOI:** 10.1186/s40359-017-0178-z

**Published:** 2017-03-20

**Authors:** Lailah Alidu, Elizabeth A. Grunfeld

**Affiliations:** 1 School of Psychology, University of Birmingham, Birmingham, England, UK; 2 Centre for Technology Enabled Health Research, Faculty of Health and Life Sciences, Coventry University, Coventry, England, UK

**Keywords:** Migrant, Health beliefs, Health behaviours Ghana, India

## Abstract

**Background:**

There is a well-established association between migration to high income countries and health status, with some groups reporting poorer health outcomes than the host population. However, processes that influence health behaviours and health outcomes across minority ethnic groups are complex and in addition, culture ascribes specific gender roles for men and women, which can further influence perspectives of health.

The aim of this study was to undertake a comparative exploration of beliefs of health among male and female Ghanaian and Indian migrants and White British participants residing in an urban area within the UK.

**Methods:**

Thirty-six participants (12 each Ghanaian, Indian and White British) were recruited through community settings and participated in a semi-structured interview focusing on participant’s daily life in the UK, perceptions of their own health and how they maintained their health. Interviews were analyzed using a Framework approach.

**Results:**

Three super ordinate themes were identified and labelled (a) beliefs about health; (b) symptom interpretation and (c) self-management and help seeking. Gender differences in beliefs and health behaviour practices were apparent across participants.

**Conclusions:**

This is the first study to undertake a comparative exploration of health beliefs among people who have migrated to the UK from Ghana and India and to compare with a local (White British) population. The results highlight a need to consider both cultural and gender-based diversity in guiding health behaviours, and such information will be useful in the development of interventions to support health outcomes among migrant populations.

## Background

The United Kingdom is a major destination for international migration [1]; between 1993 and 2011 the foreign-born population in the UK almost doubled from 3.8 million to around 7.0 million. There is a well-established association between migration to high income countries and health status, with some groups reporting poorer health outcomes over time than the host population [2, 3]. However, the relationship between migration and health is dependent upon a number of factors including ethnicity, migration status (voluntary or involuntary migration), age and gender [4]. Other factors such as long hours of work, unemployment, poorer quality housing, stress and poor command of the host country’s primary language can have detrimental impacts on migrant health outcomes [5]. Furthermore, migration influences lifestyle choices and health behaviours, with evidence of changes in dietary pattern due to challenges incorporating traditional foods as well as increased consumption of processed food [6]. As a consequence, we see an increase in chronic diseases among migrant populations, for example cardiovascular disease, stroke and Type 2 diabetes are more prevalent among people of South Asian ethnicity (e.g. Indian, Pakistani and the Bangladesh) in the UK [7, 8]. Furthermore, migrants from African countries are at a higher risk of developing chronic diseases than those that do not migrate [9]. People of African descent living in Europe have high incidence rates of stroke, diabetes and hypertension [10] and this may be attributed to differences in individual health behaviors or the socioeconomic circumstances of migrants [11].

Understanding how individuals make sense of health, and empowering communities to adopt healthy practices to prevent chronic illnesses, is an important first step to developing practical interventions to improve health outcomes among migrant populations. However, studies that have examined health beliefs and health behaviours have tended to aggregate findings from migrants across different countries of origin or have examined single groups from specific countries of origin, or ethnicities, in isolation. However, aggregation at this level does not recognize heterogeneity in beliefs and behaviours [12]. There may be the assumption that there are inter, but not intra, sharing of common beliefs and behaviours across minority ethnic groups. Africa has been described as culturally complex [13] and yet there are shared common beliefs and cultural values that center around the equal status of spiritual and physical aspects of the body in health, a strong respect for the role of elders within families and society and the role of extended kinship bonds (e.g. grandparents, aunts, uncles, cousins) in the influencing of important health-related decisions [14]. Within Ghana an estimated 70% of the population depend on complementary or alternative medicine for their healthcare [15]. Conversely, within India it is common to attribute and explain illness in terms of chance-related factors, fate or karma [16]. Furthermore, there has shown to be a reluctance to accept a diagnosis of emotional illness because it can impact on the chances of other members of the family getting married which can impact on decisions to seek help [17]. More research is needed to explore differences across cultural groups in terms of beliefs about health and health-related behaviours.

Processes that influence health behaviours and health outcomes across minority ethnic groups are complex; a range of factors influence or drive health behaviours. The patterning of such factors may, or may not, depend upon ethnicity-based groupings, and although people might be classified as the same ethnic grouping, culture ascribes specific gender roles for men and women, which can further influence perspectives of health [5]. For example, women, as a consequence of being “genetic housekeepers” or information holders within families [18],are more likely than men to cite hereditable factors as causes of conditions such as breast cancer, heart disease and arthritis [19]. Furthermore, it is increasingly recognized that it is important to understand how men “do” health and illness. It is proposed that men perceive social pressure to conform to dominant masculine gender role norms and that deviation from these norms may lead to gender role conflict [20]. However, masculine gender role ideals exist with contextual factors, such as age, class, culture and these also influence health behaviour [21]. To date much of the research in this field has been conducted with Western populations and more work is needed to understand the role of gender in moderating cultural diversity in beliefs about health. The aim of this study was to undertake a comparative exploration of beliefs of health among male and female Ghanaian and Indian migrants and White British participants residing in an urban area within the UK.

## Method

Ethical approval for the study was obtained from a the University of Birmingham Research Ethics Committee. The consolidated criteria for reporting qualitative studies (COREQ) [22] were adhered to.

### Setting

In this study, a migrant was defined as a person who was born outside of the UK but who had resided in the UK prior to recruitment for 12 months or longer either legal or illegally, voluntarily or involuntarily [1]. The study sought to recruit from an established minority group within the UK (i.e. migrants from India) and from a population who were a new or growing minority group within the UK (i.e. migrants from Ghana) as support structures within those communities might differ across these groups. According to the Office of National Statistics [23] there are around 700,000 migrants from India residing in the UK and almost 96,000 migrants from Ghana. A White British-born sample (defined as native born with both parents also being native-born British) was included to identify comparable health beliefs within the UK. Data were collected in Birmingham, which is the second most populated city in the UK with a population of over one million within the city and approximately 3.6 million within the wider metropolitan area. The area has great ethnic and racial diversity and has been labelled one of the most diverse cities in the UK, with over 170,000 new migrants each year.

### Participants and procedure

The sample was stratified by country of origin, gender and age. Participants were eligible for inclusion in the study if they were (1) aged over 18 years old, (2) were able to understand and undertake an interview in English, (3) self-identified as White British, Ghanaian or Indian, and (4) for the Ghanaian and Indian sample were born in either India or Ghana and (5) had lived in the UK for 12 months or longer prior to the interview. Potential participants were identified (face to face and through posters) through community groups (including churches and neighbourhood associations) in one major UK city. Participants were provided with information about the study and interview process and were asked to sign a consent form if they were interested in taking part. Individual face-to-face interviews were then arranged and undertaken at a location that was convenient for the participant (home or workplace) and where a private room was available to conduct the interviews. Participants were compensated for their time with a £10 shopping voucher.

### Interview schedule

An interview schedule was developed and based on an adapted Life History Interview approach [24]. This approach was chosen as it focused on the lived experiences of migrants, their personal stories and offered an in-depth account of specific experiences relating to their health beliefs. This approach was considered suitable because the research aimed to explore participants understanding of their own health. An interview guide was developed comprising open-ended questions to allow the discussion of participant-centered issues as they emerged. The interview focused on the participant’s daily life in the UK and they were asked to describe a typical day and then from this specific questions relating to their health were introduced. Specifically, these questions focused on perceptions of their own health and how they maintained their health. Interviews lasted between 45 and 80 min (mean 60 min). Interviews were audio recorded and transcribed verbatim. Transcribed data was checked against initial original recordings. To ensure confidentiality each participant was assigned a pseudonym and this was used, rather than their own name, in the analysis and presentation of findings.

### Data analysis

The transcribed data was analyzed thematically using a Framework analysis approach [25].

The Framework approach was originally developed for applied qualitative research and the approach is now widely used within the UK. The name reflects the thematic framework, which is used to classify and organise data and which is individual to each study. Following completion of all interviews, each transcript was analysed by noting relevant units of meaning and creating free codes. Free codes were then grouped into coherent themes. A matrix was developed, with emerging themes and sub themes highlighted, which facilitated the identification of themes that emerged across participants. This method allowed the data to be managed in such a way that facilitated effective interpretation and explanation of patterns and as data was organized according to case and theme, this allowed analysis across themes (thematic analysis) and within cases (case analysis). Once themes had been identified for each participant, these were integrated across participants to generate a list of superordinate themes that captured the participants’ shared experiences. The next level of analysis involved the examination of relationships and interactions between the themes. All themes emerged from the data (inductive coding) as the adapted Life Histories approach facilitated the sharing of personal and distinct experiences. Two researchers (LA and EAG) read the first four transcripts independently and undertook an initial coding process separately to identify meanings. These original codings were discussed and ordered into initial themes and these themes were then used to produce a preliminary framework that could be used to guide the subsequent analysis. The preliminary framework included all the codings and initial themes that were identified by the two researchers. The remaining analysis was undertaken by one researcher (LA) with continued discussion throughout the analysis process with the secondary author. Differences that emerged were discussed and a consensus reached between the two researchers; the differences that emerged were around the naming of new themes and whether the coding of the new text fitted with existing themes.

## Results

Forty-two people who were either approached by the researcher or contacted the researcher directly about the study were provided with information. Six people (response rate 86%) declined to participate after receiving further information about the study and reasons for non-participation included lack of time to participate or the researcher being unable to arrange an interview at a convenient time and suitable location. The sample comprised 36 participants aged between 20 and 60 years (mean age 38 years). Half of the participants (*n =* 18) were female (see Table 1). The duration of residence in the UK for both the Ghanaian and Indian participants ranged from eighteen months to ten years (mean 3.8 years). Through the analysis three super ordinate themes were identified and labelled (a) beliefs about health; (b) symptom interpretation and (c) self-management and help seeking. Cultural and gender differences were apparent in across the themes (see Table 2).

**Table 1:** Socio demographic characteristics of sample (*N =* 36)

	White British	Indian	Ghanaian
Age mean in years	38(range 20–60)	35(range 24–58)	35(range 24–60)
Gender			
Male	6	6	6
Female	6	6	6
Marital status			
Married	4	3	5
Single (never married)	8	9	7
Education			
A-level/Equivalent	3	2	2
Degree/Higher level	9	10	10
Time since arrival			
Between 1 to 5 years	-	8	8
5 years+	-	4	4

**Table 2:** Cultural and gender difference across themes

Theme	Cultural differences	Gender differences
Beliefs about health	Absence of illness (I, G) Not attending healthcare services (I, G) Good mental health (WB)	Not consciously thinking about their health (M) Absence of serious health condition (M) Monitoring food and planning meals (F) Weight and body type (F)
Symptom interpretation	Normalisation of common symptoms and illness (I/G) Normalisation of hereditary conditions (I/G)	
Self-management and help-seeking	Preference for self-management of common symptoms (I/G) Use of home remedies and traditional medicines (I/G)	Strong distinction between illness and health (M) Help-seeking for symptoms as a common behavioural response (F) Help-seeking for a range of symptoms (F)

Key: *I*, Indian, *G* Ghanaian, *WB* White British, *M* male, *F* female

### Beliefs about health

Descriptions of the meaning of health and what it means to be healthy encompassed a range of beliefs around the absence of disease, not needing to seek help from a healthcare provider as well as behaviours, such as healthy eating and adequate physical activity. In discussing the general issues about health, and why one considered him or herself to be healthy, participants often attributed health to the performance of behaviours such as making appropriate food choices and engaging in regular physical activity. There was a belief shown across all country of origin groups that diet and exercise were key to good health and that individuals needed to take personal responsibility for such behaviours.

“*I try to take a reasonable amount of fruit and vegetables. I try not to have too much caffeine, I try to be aware of how much sugary stuff I am having.” (female, Indian)*

*“I always use my dad as an example, my dad was an old soldier, military man and he was very strong, because of the military training he underwent. He always encouraged me to do lots of physical activity so I can remain healthy”* (male, Ghanaian)

#### Cultural differences

On further probing of what it meant to be healthy participants from India and Ghana spoke not only about eating a healthy diet, but also about the absence of illness and not needing to attend healthcare services. For example, a Ghanaian participant defined good health as being free from an illness that would require either hospitalization or for him to make an appointment with a GP (general practitioner). In these cases being healthy was not a consequence of personal behavioral choices but rather good luck and being able to stay away from healthcare services was frequently given as a definition of being healthy, predominantly by participants from India and Ghana but not by White British participants.

*“I think I look healthy because for the past 12 years I have never been to the hospital. The last time I was sick was when I was in the secondary school. People always wondered what was in my body because I hardly fell sick” (male, Ghanaian)*

Among the White British sample being healthy equated to eating well and having adequate physical exercises, however this group also emphasized the importance of good mental health, an aspect that was not raised by the two migrant samples.

*I suppose being healthy means being free from stress or depression and things like. That’s not only got to do with the body, but the mind as well (female, White British)*

#### Gender differences

Gender differences were apparent in terms of beliefs about what makes one healthy or how one described his or her health status. The male participants, regardless of country of origin, presented a picture of not consciously thinking about their health, unlike the women in the study who spoke about the importance of monitoring and planning foods consumed and of undertaking physical activity. Female respondents from the UK and India also spoke in terms of the role of diet and physical activity behaviors in maintaining a preferred body type. This is not so apparent among the female participants from Ghana who spoke about wanting to have “enough” body and that this was a normative expectation among their peers in Ghana. Women also recounted their experiences with their families and family history and how it had influenced them to lead a healthy lifestyle. For example, one women spoke about how her father had developed diabetes because of his unhealthy lifestyle, and how this had prompted her to start exercising and watch her diet.

Most male respondents, regardless of country of origin, described themselves as healthy and when further probed expanded to state that being healthy to them meant not having any serious health condition.

*I suppose because …….. I don’t have any, like severe health problem, as some people get pretty bad health problems. So I suppose in that regard, I regard myself as healthy (male, Indian)*

### Symptom interpretation

There were no clear gender differences in how male and female participants made attributions based on their symptoms. All participants spoke about their interpretations of particular symptoms and how this guided subsequent responses on how to prevent or manage symptoms and how previous experience influenced these symptom interpretations. Amongst all participants, common ailments, which could easily be treated with over the counter medication, or easily accessible medication, were described as “normal” and participants often indicated that they did not consider that these reflected being unhealthy or ill. For example, participants relayed that an upset stomach would be interpreted as the result of something that they had previously eaten or that a headache would be attributed to stress or too little sleep. As a consequence of these familiar symptoms participants would self-manage their condition and spoke of changing and monitoring their food choices, taking painkillers or ensuring that they had adequate rest.

However, participants from Ghana and India (not White British participants) spoke of how symptoms or illnesses that commonly occurred in one’s environment were often normalised and were not characterized as ill health. For example, participants from both India and Ghana spoke about malaria as a commonly occurring condition that was part of “normal life” in their home countries and therefore was not perceived to be an illness as such;

*“I was generally healthy. The only thing that I used to get was malaria and you know it is common when you live in Ghana” (female, Ghanaian)*

Furthermore, diseases that were hereditary, such as Type 1 diabetes, were again described as normal, particularly if they could be self-managed.

*“Diabetes is something that is common in my family, I inherited it and all I have to do is manage it. That does not make me less healthy” (male, Ghanaian*)

### Self-management and help seeking

#### Cultural differences

Beliefs about how healthy one is also influenced decisions around seeking professional help to treat or manage conditions*.* There were similarities between the reports of participants from Ghana and India, which to some degree reflected the endemic diseases in their home countries and familiar approaches to self-management. Malaria was a common endemic disease that participants from both India and Ghana talked about and that they would chose to self-manage. A rise in temperature or a fever were frequently attributed to malaria and participants had particular treatments that they would default to first such as grapefruit extract or cinnamon.

*“When I feel unwell, it is normally malaria, especially when you get a fever and you start feeling hot” (male, Indian*)

Decisions around how to manage a symptom were often influenced by the severity, or previous experience, of the symptom. Participants from Ghana and India were less likely to speak about engaging with pharmacists or healthcare providers for common symptoms and were more likely to refer to refer to home remedies or traditional medicine (such as dried or boiled roots or herbs).

*“I don’t mind any of the two, but it will depend on the situation. Western medicine can be used for severe treatment because it is more scientific. Traditional Ghanaian medicine can be used for everyday illnesses”* (male Ghanaian)

#### Gender differences

Gender differences in help-seeking decisions were apparent across all participants, regardless of country of origin. Descriptions of help-seeking were more common among the women than the men in this study. Male participants often defined being healthy as not seeking medical help. One male participant described how he had never been admitted to hospital, which he gave as evidence that he was healthy. This pattern was noticeable in other male participants who tended to make a clear distinction between illness and health.

*‘I don’t know, I can’t remember that I have ever been admitted to the hospital.….. I don’t know, I have never taken ill, seriously ill like that’ (male, Indian).*

“*I have never been to the GP here, I don’t know who or where my GP is*” *(male, Ghanaian).*

In comparison to the men in this study female participants were more likely to describe help-seeking for a range symptoms and were more likely to include help-seeking as one of their common behavioral responses to bodily changes.

*‘I feel very fine within my body, I guess if I was unhealthy I will feel sore in my body, I will probably go and see my GP” (female, Ghanaian)*

## Discussion

The aim of this study was to undertake a comparative exploration of beliefs of health among male and female Ghanaian and Indian migrants and White British participants residing in an urban area within the UK. Differences in beliefs and health behaviour practices were apparent across participants from the different countries of origin and the persistence of culturally based beliefs following migration would account to some extent for observed differences in beliefs and health practices between a host population and new migrants [5]. However, established gender differences were also apparent and women were more proactive around issues concerning their weight than men [26] which is in line with previous research reporting that women commonly opt for portion controlled or lower calorie foods [27–31] and are likely to avoid fat and consume higher amounts of fiber than men [26]. Conversely, maintaining good health is not seen to be a motivation for men’s food choice.

The analysis demonstrated a clear position that men are more likely to report that they are well because of how they attribute signs of good health. Men’s beliefs about health are shaped by societal prescription of their role and “being masculine” may involve being able to withstand challenges, conceal emotion and not disclose distress, which in turn could shape behavioral responses such as help-seeking [32, 33]. Men in our study attributed good health to the avoidance of healthcare services or not having a major illness and evidence suggests that in general men in Britain are less likely to visit their GP compared to women [34]. In Ghana it has been shown that men are three times more likely to use complementary or alternative medicine than females [35]. A recent review on delays in medical and psychological help seeking in men showed that men often delayed seeking help as they misinterpreted a symptom as insignificant and therefore not requiring professional support [36] The same review also highlighted that conformity to masculine gender role norms was an important barrier to men’s help-seeking whereby men considered medical help-seeking behaviour to be a feminine activity.

This study demonstrated that participants from Ghana and India were less likely to speak about engaging with pharmacists or healthcare providers for common symptoms. Attributing being healthy to not having a “serious condition” or to not needing to access healthcare services might reflect differing previous experiences of healthcare systems utilization. Although in the UK each person is registered with a general practitioner in their geographical area, the experiences of migrants from Ghana and India may be explained by the differences in access to health services in their respective home countries. For instance, India has a large private healthcare system although it is estimated that three quarters of the population live below the poverty line and are unable to access private healthcare; the public sector health services within India primarily focus on preventative health approaches and as such low and middle income citizens may be precluded from accessing services offered by private healthcare providers [37]. In contrast, Ghana, has a National Health Insurance System that was introduced in 2009 to provide universal access to healthcare, with each individual required to make a yearly qualifying contribution [38]. Prior to this, access to and utilisation of high quality health care was selective—graded by economic status. Coupled with a low doctor-patient ratio, this made it increasingly difficult to obtain an appointment with a doctor [39]. Recent evidence from Ghana [40] indicated an increase in preferences for alternative remedies (including traditional and faith based remedies) for treating illnesses rather than seeking an appointment with a health professional. Furthermore, minority groups in high income countries may use channels other than primary healthcare facilities (e.g. self-medication) because of minimal previous exposure to healthcare services [41]. Other issues around accessibility of healthcare services for migrant populations included inadequate knowledge of health risks, minimal understanding of public health messages and cultural and language barriers [42].

The findings should be interpreted within the limitations of this study. Firstly, the study utilized a small sample of migrants, most of whom were younger and included only two country of origin groups, which may limited the range of stories provided. Furthermore, the sample recruited into this study was highly educated and may not reflect the experiences of migrants with differing socioeconomic and immigration status. In addition, participants were recruited through community settings, including Christian churches, which may have introduced bias in the sample around the role of religion in health. Finally although the results provide novel data around the experiences of migrants in the UK, it may not possible to generalize findings to other migrant groups within other UK cities.

## Conclusions

This study is important in that it is the first study to undertake a comparison of the perceptions of health among migrants from different countries of origin (India and Ghana) living in the UK. This study adds to the existing literature indicating that migrants’ perceptions of health and engagement in health behaviours are influenced by not only cultural prescriptions formed from their home country environment but also personal and societal expectations of gender-based behaviour. Future research could consider the role of migration on perceptions of health and illness and how this impacts on help-seeking and utilization of healthcare services, which emerged during this analysis as an area likely to be impacted by beliefs about what it means to be healthy. Furthermore, cultural definitions of what it is to be healthy are essential in supporting the co-design of interventions for minority populations and the results of this study add to the call for culturally sensitive community-based interventions, which may increase engagement and lead to better health outcomes for migrant populations.
